# Screening significantly hypermethylated genes in fetal tissues compared with maternal blood using a methylated-CpG island recovery assay-based microarray

**DOI:** 10.1186/1755-8794-5-26

**Published:** 2012-06-18

**Authors:** Aihua Yin, Xiangzhong Zhang, Jing Wu, Li Du, Tianwen He, Xiaozhuang Zhang

**Affiliations:** 1 Prenatal Diagnosis Centre, Guangdong Women and Children Hospital, Guangzhou, Guangdong, 510010, China; 2 Maternal and Children Metabolic-Genetic Key Laboratory, Guangdong Women and Children Hospital, Guangzhou, Guangdong, 510010, China; 3 Department of Hematology, The Third Affiliated Hospital of Sun Yat-sen University, Guangzhou, Guangdong, 510630, China

**Keywords:** CpG islands methylation, Methyl-CpG binding protein, Microarray, Combined bisulfite restriction analysis

## Abstract

**Background:**

The noninvasive prenatal diagnosis procedures that are currently used to detect genetic diseases do not achieve desirable levels of sensitivity and specificity. Recently, fetal methylated DNA biomarkers in maternal peripheral blood have been explored for the noninvasive prenatal detection of genetic disorders. However, such efforts have covered only chromosomal aneuploidy, and fetal methylated DNA biomarkers in maternal whole blood for detecting single-gene diseases remain to be discovered.

**Methods:**

To address this issue, we systematically screened significantly hypermethylated genes in fetal tissues and compared them with maternal peripheral blood potential in an attempt to detect fetal genes in maternal peripheral blood. First, the methylated-CpG island recovery assay combined with a CpG island array was performed for four fetus-toward placental tissues and the corresponding maternal peripheral bloods. Subsequently, direct bisulfite sequencing and combined bisulfite restriction analysis (COBRA) were carried out to validate the methylation status of the hypermethylated genes that were identified by the microarray analysis.

**Results:**

Three hundred and ten significantly hypermethylated genes in the placental tissues were detected by microarray. From the top 15 hypermethylated genes detected by microarray, two were selected for sequencing validation in placental tissue and chorionic villus samples and four were selected for COBRA validation in four placental tissues, ten amniotic fluids and five chorionic villus samples. The six selected genes were confirmed to be hypermethylated in placental tissue and chorionic villus samples, but methylation of the genes could not be detected in the amniotic fluids.

**Conclusions:**

Of the many hypermethylated genes and methylation sites that were found in the fetal tissues, some have great potential to be developed into molecular markers for noninvasive prenatal diagnosis of monogenic disorders. Further clinical studies are warranted to confirm these findings.

## Background

Definitive prenatal diagnosis of genetic diseases usually requires obtaining fetal genetic material by invasive procedures such as amniocentesis, chorionic villus sampling and cordocentesis. These invasive procedures put the fetus at a small but significant risk and, therefore, are carried out only when the risk of abnormal pregnancy, estimated by maternal age, ultrasonography and other noninvasive methods, outweighs the risk of miscarriage caused by such procedures [1]. Noninvasive procedures are also available for prenatal diagnosis. These procedures use maternal periphery blood samples and quantify maternal serum proteins that are surrogate markers of the underlying genetic abnormality. Such noninvasive diagnostic procedures do not achieve the desirable levels of sensitivity and specificity, and they are not definitive [2]. For these reasons, great efforts have been made in the last decade to develop fetal molecular biomarkers for noninvasive prenatal diagnosis.

The discovery of circulating fetal free DNA in the plasma of pregnant women opened up new doors for noninvasive prenatal diagnosis [3]. Early studies have mainly examined gender and polymorphic differences between the fetus and its mother. Fetal molecular biomarkers based on differences of this kind can be used only to detect paternally inherited disease [4,5]. In 2002, Poon et al. [6] first demonstrated that methylation differences between fetal DNA and maternal whole blood DNA could potentially be used as universally applicable fetal molecular markers for noninvasive prenatal diagnosis. Since then, a number of studies have focused on the identification of differential methylation patterns between the fetus and the maternal peripheral blood [7,8]. Recently, Papageorgiou et al. [9] used methylated DNA immunoprecipitation (MeDiP) coupled with high-resolution tiling oligonucleotide array analysis to identify more than 2,000 differentially methylated regions (DMR) between female whole blood and placental DNA on chromosomes 13, 18, 21, X, and Y. In a subsequent study, they achieved the noninvasive prenatal detection of trisomy 21 by determining the fetal-specific DMRs present in the maternal peripheral blood of normal and trisomy 21 cases [10]. These studies were targeted towards the noninvasive prenatal detection of chromosomal aneuploidy and not towards single-gene genetic diseases.

In the present study, we systematically searched for potential methylation biomarkers for the noninvasive prenatal genetic detection of various inherited diseases, including single-gene genetic diseases and chromosomal aneuploidy. First, we used the methylated-CpG island recovery assay (MIRA) to capture methylated DNA. Then we performed a long oligonucleotide microarray analysis to characterize methylation patterns in the placenta at the first, second and third trimester of pregnancy. The microarray contained 60 k 60-mer probes that interrogate 4,162 genes distributed on the 23 pairs of chromosomes. The MIRA assay is based on the high affinity of the methyl-CpG-binding domain (MBD) protein which specifically recognizes methylated CpG dinucleotides [11-13]. Papageorgiou et al. [14-16] used 5-methylcytosine-specific antibodies to enrich methylated DNA; however, their method requires single-stranded DNA for recognition. Compared to this approach, the MIRA-based approach that we have proposed is more specific and more efficient in enriching methylated CpG islands on a genome-wide basis [12].

## Methods

### Samples

Seven pairs of samples of matched maternal peripheral blood and fetus-toward placental tissue were obtained from seven healthy pregnant women. Four of the pairs were randomly selected for microarray analysis and combined bisulfite restriction analysis (COBRA); the three other pairs were used to confirm the bisulfite sequencing. Maternal peripheral bloods were collected in the fasting state before delivery, and placental tissues were sampled within 30 min of delivery. Five chorionic villus and ten amniotic fluid samples were collected in first-trimester and second-trimester pregnancies respectively, to validate the methylation status of the hypermethylated genes identified using the microarray. Before the samples were used in this study, the following diseases were excluded using the retrospective clinical criteria: pre-eclampsia, gestational diabetes, intrauterine growth restriction, preterm delivery and spontaneous abortion after detection of a fetal structural or chromosomal abnormality. All samples were snap-frozen in liquid nitrogen within 6 hrs of collection and stored at −80°C until the DNA was isolated. The experiments were approved by the Ethics Committee at the Guangdong Women and Children Hospital. Informed written consent was obtained from all participants.

### MIRA microarray analysis of DNA methylation

The MIRA-based microarray analysis was performed as described previously [17] with minor modifications. Briefly, DNA was first separated from four pairs of matched fresh-frozen maternal peripheral blood and placental tissue samples using the standard phenol/chloroform technique. Then 2μg of the genomic DNA samples were sheared into 200–1000 bp fragments by Mse I (5′-TTAA) and purified to remove any fragments smaller than 100 bp using a MicroDNA Purification Kit (CoWin Biotech Company, Beijing, China) following the manufacturer’s instruction. Afterwards, the purified DNA fragments were used to enrich methylated DNA using a MBD kit (BioChain, Hayward, CA, USA) according to the manufacturer’s protocol. The MIRA-captured DNA segments were purified and amplified using GenomePlex Whole Genome Amplification Kit (Sigma) as per the supplier’s instruction. The products of whole genome amplification from the total input DNA without MBD enrichment and methylation-enriched DNA from each of the samples were labeled with cy3-dCTP and cy5-dCTP respectively, using Klenow enzyme (Takara, Dalian, China). The fluorescent dye labeled DNA was mixed and hybridized to Agilent human CpG island microarrays that were designed to interrogate 61,982 CpG dinucleotides covering 4,162 genes. After hybridization, the slides were washed and scanned on the Agilent microarray platform according to Agilent’s standard protocol. The data were extracted using Agilent Feature Extraction software. Following global mean normalization, faint probes with intensity <400 were discarded and excluded from the analysis. Unsupervised clustering analysis was performed using the Cluster software. Probes were considered positive for differential methylation between maternal blood and placental tissue if the fold changes in their MIRA/Input signaling ratios between the placental tissue and the maternal blood were >1.2 or <0.83 (q <0.05) using SAM (significance analysis of microarrays) [18]. The significant enrichment of the Gene Ontology (GO) terms associated with the hypermethylated genes was analyzed using the hypergeometric distribution in the R language software package. All microarray data have been submitted to the Gene Expression Omnibus [GEO: GSE35997].

### Validation of hypermethylated genes using direct bisulfite sequencing

Of the top 15 hypermethylated genes identified by the methylation microarray analysis, two significantly hypermethylated genes (*NR2F2* and *TFAP2C*) were selected for further validation in independent samples using direct bisulfite sequencing. Briefly, genomic DNA was bisulfite converted using the DNA methylation detection kit (BioChain, Hayward, CA, USA) as per manufacturer’s protocol. Then, 125 ng of bisulfite converted DNA was PCR amplified in 50 μL reaction mixture containing 1 × PCR Buffer (Mg2+ Plus), 200 μM of each dNTP, 0.5 μM of forward and reverse primers (primer sequences are provided in Table 1) and 1.25 units of TaKaRa Taq HS. The PCR cycling conditions were: 94°C for 5 min; 45 × (94°C for 30 s, 55°C for 30 s, 72°C for 30 s); 72°C for 7 min, and 4°C hold. The PCR products were cleaned using a MicroDNA Purification Kit. The purified PCR products were then subjected to direct sequencing in an ABI 3500xL Genetic Analyzer using the same forward primers that were used for the PCR amplification (Table 1).

**Table 1:** Primer pairs used for direct bisulfite sequencing and combined bisulfite restriction analysis of hypermethylated genes

**Gene**	**GenBank Accession No.**		**Primer (5' → 3')**
*PITX2*	NM_000325.5	F	TAGTGATAGGCGTTTCGGGTT
		R	CCACTACATACTAACAAACACTCAAAT
*TLX3*	NM_021025	F	TCGGTTGAGGATTAGAGGGATT
		R	AACGCCACCTAACCATCTATTC
*OTX2*	NM_172337.1	F	AGTTGTGTTAGGTTGAGGGAG
		R	AATCCCAAAAACCTTTTTAAA
*MNX1*	NM_001165255	F	TTTAAGAAATAGCGAGAGGGAG
		R	AAACGCTCGTAACATAATCCC
*NR2F2*	NM_001145155	F	CGTATCGTGGATTTGGAGTAGGGTATT
		R	AACAAACTCGCTAACAAATAAACRACATT
*TFAP2C*	NM_003222	F	CGAAGTGTTAGGGTTTTGTGTGT
		R	CGACCTTAAACAACAACCAAATCC

### Validation of hypermethylated genes using COBRA

COBRA was used to validate the methylation status of four of the genes (*PITX2*, *TLX3*, *OTX2* and *MNX1*) from among the top 15 hypermethylated genes [19]. The principle of the COBRA method is that the cytosine in DNA is converted to uracil by bisulfite treatment while methylated cytosine is retained as cytosine. Thus, methylated and unmethylated cytosines can be distinguished by digesting the DNA with a restriction enzyme that recognizes sequences containing CpG. For the COBRA assays, the bisulfite conversion, PCR amplification and purification were carried out as described above. The primer sequences used for the COBRA validation are listed in Table 1. The purified PCR products were digested with the *BstU* I (CG↓CG) restriction enzyme (New England Biolabs, Ipswich, MA, US) and then electrophoresed on 2% agarose gels supplemented with ethidium bromide for visualization under a UV light.

## Results

### MIRA microarray analysis of DNA methylation

**Figure 1:**
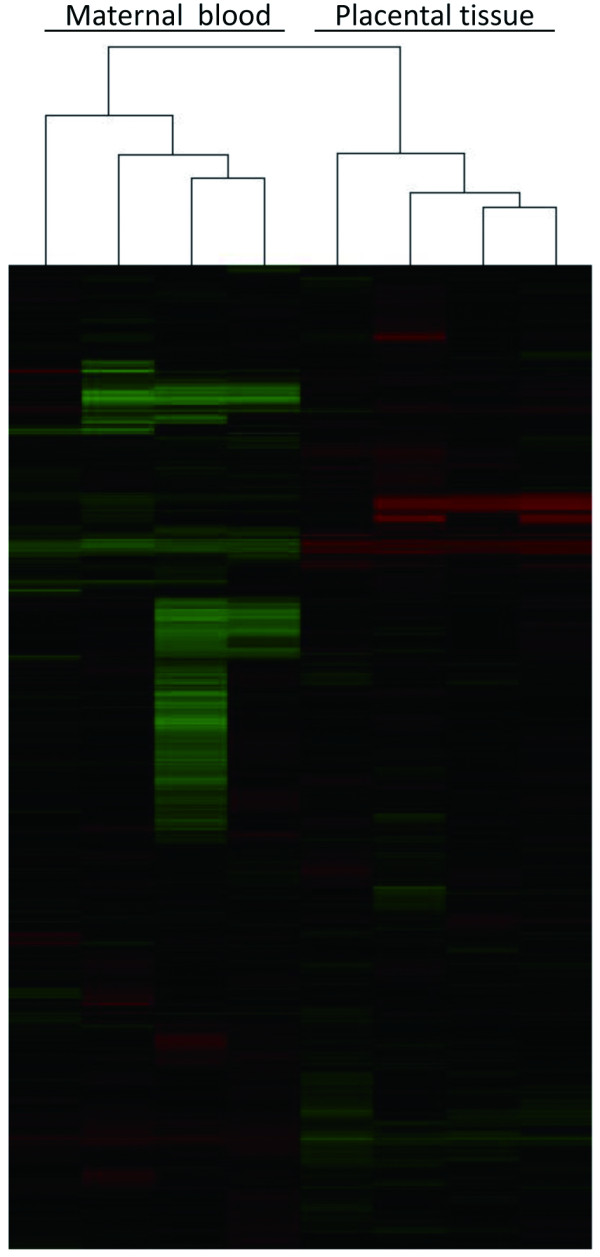
Unsupervised clustering in four pairs of matched placental tissue and maternal peripheral blood samples.

To screen hypermethylated genes in fetal tissue relative to those in maternal circulation, we used an MIRA approach to identify significantly hypermethylated genes in four placental tissue samples and the four corresponding maternal peripheral blood samples. First, we performed an unsupervised clustering analysis on 9382 probes with intensities that were greater than 400 in at least one of the samples. This analysis showed that there was distinct clustering of the maternal peripheral blood and placental tissue samples (Figure 1), suggesting that there were obvious differences of methylation between the maternal peripheral blood and the placental tissue.

The methylation difference, represented by copy number difference of MBD protein enriched DNA in MIRA approach, is similar to the DNA copy number difference in array-based comparative genomic hybridization (aCGH) and is quite different from the gene expression for one copy of DNA expresses various copies of mRNA. In the well-established aCGH method, the cutoff for detecting DNA copy differences is usually set to be >1.25 of probe ratio value [20,21]. This cutoff is lower than the one used in mRNA expression profiling analysis where the alteration ratio is usually set to be >2.0. In the present study, SAM analysis was performed based on the cutoff criteria that fold changes in MIRA/Input signaling ratios between placental tissues versus maternal bloods were >1.2 or <0.83 (q <0.05). We detected 3,774 positive probes corresponding to 783 differentially methylated genes; of these, 310 genes had at least two positive probes with fold changes in the MIRA/Input signaling ratios between placental tissues and maternal bloods that were above 1.2 (q <0.05). The 310 genes were selected as hypermethylated genes (Additional file 1: Table S 1). The top 15 hypermethylated genes had more than ten positive probes and are listed in Table 2.

**Table 2:** Top 15 hypermethylated genes identified using the MIRA-based microarray

**Gene**	**Changed probes**	**Average ratio^a^**	**Position^b^**	**Gene description**	**Phenotype MIM Accession No.^c^**
*PITX2*	27	3.19	I and D	Paired-like homeodomain 2	180500, 137600, 604229, 180550
*PAX6*	20	2.34	P and I	Paired box 6	106210, 604219, 120430, 120200, 136520, 206700, 148190, 165550, 604229
*NR2F2*	17	3.22	P, I and D	Nuclear receptor subfamily 2, group F, member 2	
*MNX1*	15	2.50	P, I and D	Motor neuron and pancreas homeobox 1	176450
*TLX3*	15	3.69	P and D	T-cell leukemia homeobox 3	
*PAX9*	15	2.33	P and I	Paired box 9	604625
*SALL1*	15	2.27	P, I and D	Sal-like 1 (Drosophila)	107480
*MAD1L1*	14	3.18	I	MAD1 mitotic arrest deficient-like 1 (yeast)	176807
*TBX3*	14	4.50	P, I and D	T-box 3	181450
*HLX*	14	2.01	P, I and D	H2.0-like homeobox	
*PDX1*	13	2.88	P, I and D	Pancreatic and duodenal homeobox 1	606392, 260370, 125853
*TFAP2C*	13	4.78	P	Transcription factor AP-2 gamma	
*SIX1*	12	3.13	P, I and D	SIX homeobox 1	608389, 605192
*SIM1*	12	2.98	P and I	Single-minded homolog 1 (Drosophila)	601665
*OTX2*	12	3.39	P and D	Orthodenticle homeobox 2	610125, 613986

Several probes for each gene are present on the microarray. ^a^The average ratio of the probe density of the positive probe in placental tissue to that in the maternal peripheral blood calculated using the SAM software. ^b^The letters in this column refer to different regions of the gene: P, promoter; I, inside; D, downstream. ^c^The phenotype MIM Accession Number is from the OMIM database (http://www.ncbi.nlm.nih.gov/omim).

**Figure 2:**
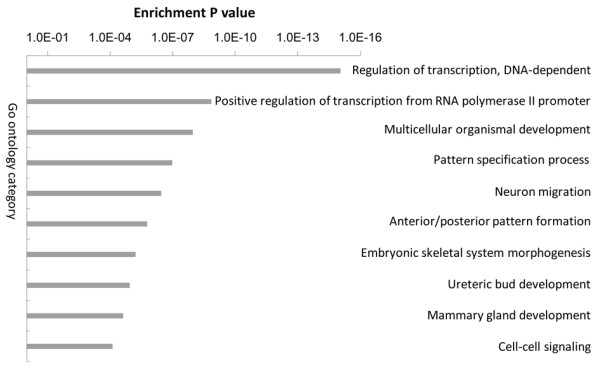
Gene ontology categories enriched in differentially methylated genes between placental tissues and maternal peripheral blood.

The significant enrichment analysis of the GO terms for the differentially methylated genes demonstrated that the 783 differentially methylated genes were involved in many important biological processes such as regulation of transcription and organismal development (Figure 2).

### Validation of hypermethylated genes using direct bisulfite sequencing

**Figure 3:**
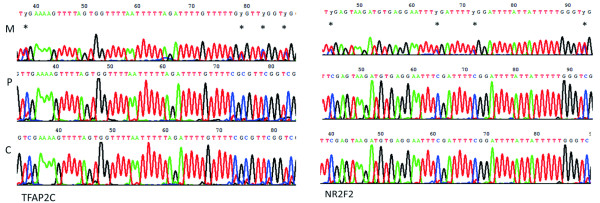
**CpG methylations of *TFAP2C *and *NR2F2 *measured using direct bisulfite sequencing.** Methylated CpGs were observed at nucleotide positions −1516(38), −1475(79), −1471(83) and −1464(87) in *TFAP2C*, and at −2467(44), −2446(65), −2439(72) and −2418(93) in *NR2F2*. Y indicates either T or C.

Bisulfite sequencing allows the methylation analysis of the cytosine residues in a given sequence. Two protocols have been employed for bisulfite sequencing: cloning-based sequencing and direct PCR sequencing. Cloning-based sequencing is very useful in determining the pattern of mosaic methylation of individual molecules. However, it is labor-intensive and time-consuming because it requires the cloning of the PCR product before sequencing and usually the sequences of at least ten individual clones are required to provide an accurate estimate of methylation in the population of molecules. Direct PCR sequencing only provides the averaged methylation status in a population of molecules but this method is convenient for the rapid assessment of global methylation levels in a population of molecules. In this study, direct bisulfite sequencing was performed to validate the reliability of the methylation microarray analysis. Two genes, *TFAP2C* and *NR2F2*, from the top 15 hypermethylated genes identified by MIRA analysis were selected for methylation validation in maternal peripheral blood, placental tissue and chorionic villus samples. The sequencing results indicated that *TFAP2C* and *NR2F2* were hypomethylated at multiple CpGs sites in maternal peripheral blood samples and hypermethylated at multiple CpGs sites in the placental tissue and chorionic villus samples (Figure 3) of fetal origin.

### Validation of hypermethylated genes using COBRA

**Figure 4:**
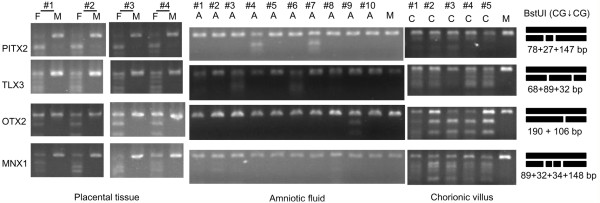
**Methylations of *PITX2, TLX3*, *OTX2 *and *MNX1 *genes validated using the combined bisulfite restriction analysis.** Bisulfite converted genomic DNA was amplified with gene-specific primers followed by digestion with a methylation sensitive enzyme *BstU*I (CG↓CG). Methylation status was measured in placental tissue (P), maternal peripheral blood (M) and chorionic villus (C). The expected enzyme-digested pattern of the PCR products in which CpG islands were fully methylated was depicted on right side.

COBRA is a sensitive and convenient assay to measure DNA methylation status at specific gene loci in small amounts of genomic DNA [22,23]. COBRA has been widely used for confirming methylation results obtained by microarray analysis [12,24]. Here the COBRA assay was used to validate the reliability of the methylation microarray analysis and to screen for fetal methylation biomarkers. Four genes from the top 15 hypermethylated genes were selected for validation in four pairs of matched placental tissue and maternal peripheral blood samples. The results in Figure 3 show that *PITX2*, *TLX3*, *OTX2* and *MNX1* were all methylated in placental tissues but not in maternal peripheral bloods. Because the placental tissues are at a late stage of fetus development, we also measured the methylation status of these four genes in ten amniotic fluid and five chorionic villus samples using a COBRA assay to assess the methylation status at the early and middle stages of fetus development. The results showed that *PITX2*, *TLX3*, *OTX2* and *MNX1* were also methylated in all the chorionic villus samples tested (Figure 4), but were not methylated in the majority of the tested amniotic fluid samples.

## Discussion

The MIRA microarray does not require restriction endonuclease, antibody or bisulfite treatment of the genomic DNA and, therefore, offers many advantages over existing methods for the genome-wide screening of DNA methylation. MIRA microarrays have been used to identify candidate methylation biomarker for cancer diagnosis [12,25]. In this study, we applied this approach to seek candidate biomarkers for noninvasive prenatal diagnosis. Currently, noninvasive prenatal screening typically involves a combination of ultrasound tests and the measurement of non-specific maternal serum markers. These screening tests are limited to trisomies of chromosomes 21 and 18 and do not reliably diagnose or exclude these types of abnormalities. The discovery of fetal DNA in maternal plasma opened new doors for non-invasive prenatal diagnosis. However, the presence of background maternal DNA interferes with the analysis of fetal DNA which usually constitutes less than 10% of the total circulating free DNA in early pregnancy [26]. This has posed significant technical hurdles for the detection of fetal genetic loci that are not completely absent from the maternal genome using current PCR-based approaches [27]. To overcome this problem, the differential methylation between placentally and maternally derived cell-free DNA sequences has been investigated [28]. Many studies have shown that these epigenetic differences may serve as potential fetal molecular markers for noninvasive prenatal diagnosis [7,8]. However, only a limited number of genomic regions have been identified or tested so far and the majority of studies have focused only on fetal chromosomal aneuploidy detection, for example, of chromosomes 21 and 18. Here, we aimed to identify methylation biomarkers for the prenatal diagnosis of not only fetal chromosomal aneuploidies but also of monogenic diseases at a genome-wide level.

Promoter methylation plays important roles in regulating gene expression both in development and in human disease and, so far, DNA methylation studies have mainly been focused on the promoter regions of genes. Recently, methylation of the gene body (sometimes called intragenic methylation) has been reported to play a role in transcriptional regulation and efficiency [29] and intragenic methylation is attracting increasing attention. Therefore, in this study, we investigated the hypermethylation of genes based on the methylation status of both the promoter and the gene body.

Large differences were observed in methylation patterns between maternal peripheral blood and placental tissue. Further analysis based on GO terms revealed that many of the differentially methylated genes were involved in regulation of transcription and multicellular organismal. This result might suggest that the differentially methylated genes contribute to the control of gene expression during embryonic development. It is well known that DNA methylation changes during embryonic development are frequent events that play major roles in regulating gene expression and other developmental processes. It is worth noting that differentially methylated genes were involved in mammary gland development (Figure 2). This finding suggested that methylation may play major roles in regulating lactation.

The bisulfite sequencing and COBRA assays both confirmed that the observed genes were hypermethylated in placental tissue and chorionic villus; however, the DNA methylation was unobvious in the amniotic fluid samples. The placental tissue samples, obtained immediately after delivery, are from a late-stage placenta and the chorionic villus tissue samples are from an early-stage placenta, the amniotic fluid is not a placental tissue. The cells in amniotic fluid are an admixture of various cells from various fetal tissues, mainly fibroblasts, epithelial cells and amniocytes that are shed from the fetus surface. Therefore, we concluded that the observed methylation differences in these samples may reflect tissue specificity rather than developmental specificity.

Previous studies have shown that the circulating fetal DNA in maternal peripheral blood are mainly from the placenta [30] because the placenta is the only channel for nutrient transport between mother and fetus. The DNA in different tissues carries the same sequence information, no matter whether the sequences are methylated or not. Therefore, the detection of fetal methylated DNAs in maternal peripheral blood will yield fetal information about genetic variations that may be useful for the diagnosis of fetal genetic diseases. Furthermore, it is convenient and feasible to discriminate between fetal methylated DNA and maternal nonmethylated DNA in maternal peripheral blood based on differences in their methylation patterns. Thus, the observed methylation differences of disease-associated genes between placental tissue and maternal peripheral blood provide a foundation for developing novel methods for the detection of fetal genes in maternal peripheral blood.

We identified a large number of hypermethylated genes in fetal tissues; most of these genes have been recorded in the Online Mendelian Inheritance in Man (OMIM) database (http://www.ncbi.nlm.nih.gov/omim) where the relationship between abnormalities in these genes and diseases has been defined. For example, mutations in *PITX2*, a homeobox gene, are known to contribute to Axenfeld-Rieger syndrome (ARS), an autosomal-dominant developmental disorder [31,32]. The hypermethylated genes have great potential to be developed into molecular markers for noninvasive prenatal diagnosis of monogenic disorders. In a future study, we will use the MBD protein to enrich fetal hypermethylated DNA fragments in maternal peripheral blood and further explore the feasibility of using these hypermethylated genes as biomarkers for noninvasive prenatal diagnosis in large samples.

## Conclusions

In this study we identified a lot of hypermethylated genes and methylation sites in fetal tissues. Some of the hypermethylated genes have great potential to be developed into molecular markers for noninvasive prenatal diagnosis of monogenic disorders. Further clinical studies are warranted to confirm these findings.

## Abbreviations

COBRA: Combined bisulfite restriction analysis; MIRA: Methylated-CpG island recovery assay.

## Competing interest

The authors declare that they have no competing interests.

## Authors’ contributions

XZ Zhang, AH Yin and XZ Zhang defined the research theme. AH Yin, XZ Zhang, J Wu, L Du and TW He performed the experimental work and organized the data. AH Yin and XZ Zhang designed experiments, interpreted data and drafted the manuscript. XZ Zhang critically reviewed the manuscript and provided concepts. All authors read and approved the final manuscript.

## Pre-publication history

The pre-publication history for this paper can be accessed here:

http://www.biomedcentral.com/1755-8794/5/26/prepub

## Supplementary Material

Additional file 1 **Table S1.** List of the hypermethylated genes in placental tissue identified by MIRA based microarray. Click here for file
