# Supplementary material for: Screening significantly hypermethylated genes in fetal tissues compared with maternal blood using a methylated-CpG island recovery assay-based microarray
Source: BMC Med Genomics. 2012 Jun 18;5:26. doi: 10.1186/1755-8794-5-26 (PMC3534415; doi:10.1186/1755-8794-5-26)
Supplement: Additional file 1 — Table S1. List of the hypermethylated genes in placental tissue identified by MIRA based microarray. [file 1755-8794-5-26-S1.pdf]

Additional material

Table S1. List of the hypermethylated genes in placental tissue identified by MIRA based microarray

Gene	Total number of Probes	Number of upgraded probes	Gene description
PITX2	83	27	Paired-like homeodomain 2
PAX6	145	20	Paired box 6
NR2F2	96	17	Nuclear receptor subfamily 2, group F, member 2
MNX1	33	15	Motor neuron and pancreas homeobox 1
TLX3	66	15	T-cell leukemia homeobox 3
PAX9	58	15	Paired box 9
SALL1	75	15	Sal-like 1 (Drosophila)
MAD1L1	186	14	MAD1 mitotic arrest deficient-like 1 (yeast)
TBX3	87	14	T-box 3
HLX	72	14	H2.0-like homeobox
PDX1	58	13	Pancreatic and duodenal homeobox 1
TFAP2C	77	13	Transcription factor AP-2 gamma (activating enhancer binding protein 2 gamma)
SIX1	70	12	SIX homeobox 1
SIM1	59	12	Single-minded homolog 1 (Drosophila)
OTX2	46	12	Orthodenticle homeobox 2
CBX4	100	10	Chromobox homolog 4 (Pc class homolog, Drosophila)
TFAP2A	98	10	Transcription factor AP-2 alpha (activating enhancer binding protein 2 alpha)
OSR1	60	10	Odd-skipped related 1 (Drosophila)
PTGER4	23	10	Prostaglandin E receptor 4 (subtype EP4)
ONECUT2	78	10	One cut homeobox 2
ADRA1A	28	10	Adrenergic, alpha-1A-, receptor
HOXA6	39	10	Homeobox A6
POU4F2	44	9	POU class 4 homeobox 2
TBX5	67	9	T-box 5
NKX2-2	146	9	NK2 homeobox 2
TLX1	57	9	T-cell leukemia homeobox 1
HAND1	46	8	Heart and neural crest derivatives expressed 1
MSX1	85	8	Msh homeobox 1
WT1	52	8	Wilms tumor 1
SLC12A7	93	8	Solute carrier family 12 (potassium/chloride transporters), member 7
FOXC1	78	8	Forkhead box C1
NR5A2	46	8	Nuclear receptor subfamily 5, group A, member 2
PHOX2B	31	8	Paired-like homeobox 2b
CCND2	59	7	Cyclin D2

OLIG2	82	7	Oligodendrocyte lineage transcription factor 2
DLX4	61	7	Distal-less homeobox 4
PKD1	130	7	Polycystic kidney disease 1 (autosomal dominant)
TFAP2B	47	7	Transcription factor AP-2 beta (activating enhancer binding protein 2 beta)
PITX1	52	7	Paired-like homeodomain 1
MEIS1	79	7	Meis homeobox 1
LMX1A	56	7	LIM homeobox transcription factor 1, alpha
ROR2	51	7	Receptor tyrosine kinase-like orphan receptor 2
PDGFRA	75	7	Platelet-derived growth factor receptor, alpha polypeptide
WIT1	29	7	Wilms tumor upstream neighbor 1
EN2	109	6	Engrailed homeobox 2
SOX1	65	6	SRY (sex determining region Y)-box 1
TSSC1	44	6	Tumor suppressing subtransferable candidate 1
SOX9	71	6	SRY (sex determining region Y)-box 9
NR4A3	73	6	Nuclear receptor subfamily 4, group A, member 3
BAI2	43	6	Brain-specific angiogenesis inhibitor 2
WNT5A	66	6	Wingless-type MMTV integration site family, member 5A
HSF4	53	6	Heat shock transcription factor 4
GAD2	31	6	Glutamate decarboxylase 2 (pancreatic islets and brain, 65kda)
GATA2	85	6	GATA binding protein 2
FOXF2	55	6	Forkhead box F2
LHX2	103	6	LIM homeobox 2
HOXB13	22	6	Homeobox B13
CD8A	34	6	CD8a molecule
DLEU2	51	6	Deleted in lymphocytic leukemia 2 (non-protein coding)
GRID2	20	6	Glutamate receptor, ionotropic, delta 2
HOXA9	37	6	Homeobox A9
HOXD3	61	6	Homeobox D3
CUL4A	14	6	Cullin 4A
TOLLIP	50	6	Toll interacting protein
GNG4	33	5	Guanine nucleotide binding protein (G protein), gamma 4
ADCYAP1	56	5	Adenylate cyclase activating polypeptide 1 (pituitary)
FOXA1	70	5	Forkhead box A1
AXIN1	49	5	Axin 1
PTRF	34	5	Polymerase I and transcript release factor
IRS1	37	5	Insulin receptor substrate 1
FOXB1	69	5	Forkhead box B1
EPHA7	24	5	EPH receptor A7
BCL2L11	52	5	BCL2-like 11 (apoptosis facilitator)
AVPR1A	17	5	Arginine vasopressin receptor 1A
SLC30A3	26	5	Solute carrier family 30 (zinc transporter), member 3

CYP24A1	17	5	Cytochrome P450, family 24, subfamily A, polypeptide 1
CYP1B1	35	5	Cytochrome P450, family 1, subfamily B, polypeptide 1
HOXA1	28	5	Homeobox A1
KCNA1	28	5	Potassium voltage-gated channel, shaker-related subfamily, member 1 (episodic ataxia with myokymia)
TLX2	36	5	T-cell leukemia homeobox 2
FGF12	34	5	Fibroblast growth factor 12
NKX2-1	25	5	NK2 homeobox 1
TAL1	34	5	T-cell acute lymphocytic leukemia 1
GAD1	64	5	Glutamate decarboxylase 1 (brain, 67kda)
GRIA2	17	5	Glutamate receptor, ionotropic, AMPA 2
RORA	46	4	RAR-related orphan receptor A
FOXJ1	29	4	Forkhead box J1
KLF9	45	4	Kruppel-like factor 9
SLIT2	37	4	Slit homolog 2 (Drosophila)
ISL1	44	4	ISL LIM homeobox 1
NKD1	19	4	Naked cuticle homolog 1 (Drosophila)
FGF8	18	4	Fibroblast growth factor 8 (androgen-induced)
HOXC5	26	4	Homeobox C5
RPS6KA2	48	4	Ribosomal protein S6 kinase, 90kda, polypeptide 2
CCNA1	12	4	Cyclin A1
DLX2	74	4	Distal-less homeobox 2
SNTG1	12	4	Syntrophin, gamma 1
GNA11	43	4	Guanine nucleotide binding protein (G protein), alpha 11 (Gq class)
ZFP36L2	30	4	Zinc finger protein 36, C3H type-like 2
HOXB8	42	4	Homeobox B8
NPY	17	4	Neuropeptide Y
GRIA3	16	4	Glutamate receptor, ionotrophic, AMPA 3
DLL4	72	4	Delta-like 4 (Drosophila)
CGB7	13	4	Chorionic gonadotropin, beta polypeptide 7
A2BP1	50	4	Ataxin 2-binding protein 1
HTR1B	18	4	5-hydroxytryptamine (serotonin) receptor 1B
POU4F3	40	4	POU class 4 homeobox 3
MYF6	9	4	Myogenic factor 6 (herculin)
TCF7	11	4	Transcription factor 7 (T-cell specific, HMG-box)
HOXB2	36	4	Homeobox B2
AVP	20	4	Arginine vasopressin
FLI1	47	3	Friend leukemia virus integration 1
GFRA2	32	3	GDNF family receptor alpha 2
CTDP1	88	3	CTD (carboxy-terminal domain, RNA polymerase II, polypeptide A) phosphatase, subunit 1
PTBP1	66	3	Polypyrimidine tract binding protein 1
SKI	97	3	V-ski sarcoma viral oncogene homolog (avian)

LMX1B	129	3	LIM homeobox transcription factor 1, beta
CDKN2A	33	3	Cyclin-dependent kinase inhibitor 2A (melanoma, p16, inhibits CDK4)
CARD11	17	3	Caspase recruitment domain family, member 11
DYSF	18	3	Dysferlin, limb girdle muscular dystrophy 2B (autosomal recessive)
FOXA2	42	3	Forkhead box A2
COL18A1	75	3	Collagen, type XVIII, alpha 1
CDX2	58	3	Caudal type homeobox 2
SHH	62	3	Sonic hedgehog homolog (Drosophila)
CYP2E1	22	3	Cytochrome P450, family 2, subfamily E, polypeptide 1
GRIN2A	43	3	Glutamate receptor, ionotropic, N-methyl D-aspartate 2A
GBX2	75	3	Gastrulation brain homeobox 2
IRF4	19	3	Interferon regulatory factor 4
HIC1	52	3	Hypermethylated in cancer 1
PTPRF	39	3	Protein tyrosine phosphatase, receptor type, F
MAG	14	3	Myelin associated glycoprotein
SLC6A5	33	3	Solute carrier family 6 (neurotransmitter transporter, glycine), member 5
CHRNE	10	3	Cholinergic receptor, nicotinic, epsilon
NR2F1	23	3	Nuclear receptor subfamily 2, group F, member 1
ATOH1	34	3	Atonal homolog 1 (Drosophila)
NEUROG3	48	3	Neurogenin 3
RARB	5	3	Retinoic acid receptor, beta
PCDHGC5	20	3	Protocadherin gamma subfamily C, 5
NEDD4L	37	3	Neural precursor cell expressed, developmentally down-regulated 4-like
ST8SIA1	21	3	ST8 alpha-N-acetyl-neuraminide alpha-2,8-sialyltransferase 1
MAP2K2	34	3	Mitogen-activated protein kinase kinase 2
HLA-A	10	3	Major histocompatibility complex, class I, A
TPM1	33	3	Tropomyosin 1 (alpha)
SLC19A1	34	3	Solute carrier family 19 (folate transporter), member 1
PPP1CA	10	3	Protein phosphatase 1, catalytic subunit, alpha isoform
TFDP1	27	3	Transcription factor Dp-1
HOXD10	24	3	Homeobox D10
PDGFA	47	3	Platelet-derived growth factor alpha polypeptide
MEOX2	11	3	Mesenchyme homeobox 2
KCNJ3	22	3	Potassium inwardly-rectifying channel, subfamily J, member 3
VSX1	39	3	Visual system homeobox 1
SSTR1	35	3	Somatostatin receptor 1
NR2F6	22	3	Nuclear receptor subfamily 2, group F, member 6
PAX3	28	3	Paired box 3

GFRA1	37	3	GDNF family receptor alpha 1
RBP3	6	3	Retinol binding protein 3, interstitial
HOXC8	20	3	Homeobox C8
KCNIP2	31	3	Kv channel interacting protein 2
PTK6	11	3	PTK6 protein tyrosine kinase 6
LTK	22	3	Leukocyte receptor tyrosine kinase
WNT5B	22	3	Wingless-type MMTV integration site family, member 5B
HOXB7	23	3	Homeobox B7
GHSR	18	3	Growth hormone secretagogue receptor
KCNJ2	10	2	Potassium inwardly-rectifying channel, subfamily J, member 2
MATK	20	2	Megakaryocyte-associated tyrosine kinase
NR4A2	60	2	Nuclear receptor subfamily 4, group A, member 2
PDLIM7	18	2	PDZ and LIM domain 7 (enigma)
FOXK2	36	2	Forkhead box K2
KCNC1	26	2	Potassium voltage-gated channel, Shaw-related subfamily, member 1
CACNA1E	29	2	Calcium channel, voltage-dependent, R type, alpha 1E subunit
CNTFR	38	2	Ciliary neurotrophic factor receptor
ATP1A1	17	2	Atpase, Na+/K+ transporting, alpha 1 polypeptide
CSRP1	14	2	Cysteine and glycine-rich protein 1
ZBTB16	9	2	Zinc finger and BTB domain containing 16
CSNK1G2	56	2	Casein kinase 1, gamma 2
SHANK1	62	2	SH3 and multiple ankyrin repeat domains 1
ZNF217	9	2	Zinc finger protein 217
BMP4	47	2	Bone morphogenetic protein 4
IRS2	33	2	Insulin receptor substrate 2
CCK	20	2	Cholecystokinin
ZIC3	54	2	Zic family member 3 (odd-paired homolog, Drosophila)
MYC	42	2	V-myc myelocytomatosis viral oncogene homolog (avian)
PCSK9	17	2	Proprotein convertase subtilisin/kexin type 9
DHRS3	27	2	Dehydrogenase/reductase (SDR family) member 3
BLVRB	12	2	Biliverdin reductase B (flavin reductase (NADPH))
TOPORS	22	2	Topoisomerase I binding, arginine/serine-rich
RERE	32	2	Arginine-glutamic acid dipeptide (RE) repeats
GDNF	41	2	Glial cell derived neurotrophic factor
INPP4B	20	2	Inositol polyphosphate-4-phosphatase, type II, 105kda
MOV10L1	19	2	Mov10l1, Moloney leukemia virus 10-like 1, homolog (mouse)
TACC1	18	2	Transforming, acidic coiled-coil containing protein 1
NFIB	35	2	Nuclear factor I/B
SORBS1	10	2	Sorbin and SH3 domain containing 1
FZD5	35	2	Frizzled homolog 5 (Drosophila)

JUNB	35	2	Jun B proto-oncogene
SMARCA4	36	2	SWI/SNF related, matrix associated, actin dependent regulator of chromatin, subfamily a, member 4
SLC43A1	29	2	Solute carrier family 43, member 1
COL2A1	25	2	Collagen, type II, alpha 1
IGF1R	51	2	Insulin-like growth factor 1 receptor
PIK3R2	35	2	Phosphoinositide-3-kinase, regulatory subunit 2 (beta)
FOXF1	72	2	Forkhead box F1
TERT	81	2	Telomerase reverse transcriptase
SOCS1	24	2	Suppressor of cytokine signaling 1
P2RX2	18	2	Purinergic receptor P2X, ligand-gated ion channel, 2
MMP17	44	2	Matrix metallopeptidase 17 (membrane-inserted)
GRM7	16	2	Glutamate receptor, metabotropic 7
RGS12	38	2	Regulator of G-protein signaling 12
JUP	17	2	Junction plakoglobin
HIPK2	12	2	Homeodomain interacting protein kinase 2
FOSB	35	2	FBJ murine osteosarcoma viral oncogene homolog B
DCHS1	18	2	Dachsous 1 (Drosophila)
POMC	19	2	Proopiomelanocortin
JUN	44	2	Jun oncogene
GATA4	85	2	GATA binding protein 4
SMAD6	10	2	SMAD family member 6
KCNMA1	20	2	Potassium large conductance calcium-activated channel, subfamily M, alpha member 1
UBE2G1	14	2	Ubiquitin-conjugating enzyme E2G 1 (UBC7 homolog, yeast)
SOX4	39	2	SRY (sex determining region Y)-box 4
POU3F4	18	2	POU class 3 homeobox 4
NOS1	18	2	Nitric oxide synthase 1 (neuronal)
ROCK2	12	2	Rho-associated, coiled-coil containing protein kinase 2
HRK	25	2	Harakiri, BCL2 interacting protein (contains only BH3 domain)
INTS6	14	2	Integrator complex subunit 6
ATF3	48	2	Activating transcription factor 3
KIAA0020	5	2	Kiaa0020
DSTN	14	2	Destrin (actin depolymerizing factor)
ZNF232	35	2	Zinc finger protein 232
PTP4A2	16	2	Protein tyrosine phosphatase type IVA, member 2
RHEB	16	2	Ras homolog enriched in brain
POLR2I	16	2	Polymerase (RNA) II (DNA directed) polypeptide I, 14.5kda
THRB	20	2	Thyroid hormone receptor, beta (erythroblastic leukemia viral (v-erb-a) oncogene homolog 2, avian)
IGFALS	28	2	Insulin-like growth factor binding protein, acid labile

			subunit
PDXK	26	2	Pyridoxal (pyridoxine, vitamin B6) kinase
CUGBP2	21	2	CUG triplet repeat, RNA binding protein 2
GALR1	27	2	Galanin receptor 1
TBXA2R	23	2	Thromboxane A2 receptor
FADS2	22	2	Fatty acid desaturase 2
ENAH	14	2	Enabled homolog (Drosophila)
RGS19	16	2	Regulator of G-protein signaling 19
YWHAZ	25	2	Tyrosine 3-monooxygenase/tryptophan 5-monooxygenase activation protein, zeta polypeptide
SCHIP1	21	2	Schwannomin interacting protein 1
KCNB2	7	2	Potassium voltage-gated channel, Shab-related subfamily, member 2
KCNJ9	16	2	Potassium inwardly-rectifying channel, subfamily J, member 9
ELAVL2	32	2	ELAV (embryonic lethal, abnormal vision, Drosophila)-like 2 (Hu antigen B)
LTB	8	2	Lymphotoxin beta (TNF superfamily, member 3)
CHAT	15	2	Choline acetyltransferase
ZNF167	4	2	Zinc finger protein 167
SLC16A2	30	2	Solute carrier family 16, member 2 (monocarboxylic acid transporter 8)
NEUROD1	28	2	Neurogenic differentiation 1
CSNK1D	49	2	Casein kinase 1, delta
S100A6	10	2	S100 calcium binding protein A6
EOMES	50	2	Eomesodermin homolog (Xenopus laevis)
MFI2	29	2	Antigen p97 (melanoma associated) identified by monoclonal antibodies 133.2 and 96.5
PLS3	15	2	Plastin 3 (T isoform)
FBN1	20	2	Fibrillin 1
SH3GL1	13	2	SH3-domain GRB2-like 1
STAT1	13	2	Signal transducer and activator of transcription 1, 91kda
EPHB1	19	2	EPH receptor B1
EFEMP1	9	2	EGF-containing fibulin-like extracellular matrix protein 1
YBX1	12	2	Y box binding protein 1
STK10	19	2	Serine/threonine kinase 10
NR3C2	30	2	Nuclear receptor subfamily 3, group C, member 2
RTN4	15	2	Reticulon 4
EGR4	35	2	Early growth response 4
HTR1A	18	2	5-hydroxytryptamine (serotonin) receptor 1A
PML	13	2	Promyelocytic leukemia
DSC3	15	2	Desmocollin 3
CHP2	11	2	Calcineurin B homologous protein 2
FAM8A1	10	2	Family with sequence similarity 8, member A1

TULP1	13	2	Tubby like protein 1
TNK1	13	2	Tyrosine kinase, non-receptor, 1
SLC5A5	20	2	Solute carrier family 5 (sodium iodide symporter), member 5
ADRA2B	14	2	Adrenergic, alpha-2B-, receptor
VPS4B	12	2	Vacuolar protein sorting 4 homolog B (S. Cerevisiae)
IL1R2	3	2	Interleukin 1 receptor, type II
SYMPK	14	2	Symplekin
WNT2	21	2	Wingless-type MMTV integration site family member 2
HSPA5	17	2	Heat shock 70kda protein 5 (glucose-regulated protein, 78kda)
LRP8	29	2	Low density lipoprotein receptor-related protein 8, apolipoprotein e receptor
NTN1	39	2	Netrin 1
RAB4A	4	2	RAB4A, member RAS oncogene family
ADK	13	2	Adenosine kinase
SMPD3	36	2	Sphingomyelin phosphodiesterase 3, neutral membrane (neutral sphingomyelinase II)
KCNK15	15	2	Potassium channel, subfamily K, member 15
MOBP	6	2	Myelin-associated oligodendrocyte basic protein
NID2	22	2	Nidogen 2 (osteonidogen)
PAX8	20	2	Paired box 8
RB1	26	2	Retinoblastoma 1
CD70	10	2	CD70 molecule
RUNX2	27	2	Runt-related transcription factor 2
ADORA2B	19	2	Adenosine A2b receptor
ARHGEF1	26	2	Rho guanine nucleotide exchange factor (GEF) 1
TTYH1	16	2	Tweety homolog 1 (Drosophila)
BRD2	33	2	Bromodomain containing 2
MGAT3	39	2	Mannosyl (beta-1,4-)-glycoprotein beta-1,4-N-acetylglucosaminyltransferase
BNIP3L	10	2	BCL2/adenovirus E1B 19kda interacting protein 3-like
CNOT3	12	2	CCR4-NOT transcription complex, subunit 3
DCC	15	2	Deleted in colorectal carcinoma
FOXO3	25	2	Forkhead box O3
ASCL1	31	2	Achaete-scute complex homolog 1 (Drosophila)
PMP22	22	2	Peripheral myelin protein 22
TLR2	11	2	Toll-like receptor 2
GPC6	15	2	Glypican 6
PDK3	10	2	Pyruvate dehydrogenase kinase, isozyme 3
RECQL4	13	2	Recq protein-like 4
TRPS1	15	2	Trichorhinophalangeal syndrome I
E2F3	17	2	E2F transcription factor 3
NTN3	27	2	Netrin 3

PTGIS	7	2	Prostaglandin I2 (prostacyclin) synthase
PLXNA3	31	2	Plexin A3
